# Obtaining psychological embeddings through joint kernel and metric learning

**DOI:** 10.3758/s13428-019-01285-3

**Published:** 2019-08-20

**Authors:** Brett D. Roads, Michael C. Mozer

**Affiliations:** 1 Department of Computer Science, University of Colorado Boulder, Boulder, CO 80309-0430 USA; 2 Present Address: Department Experimental Psychology, University College London, 26 Bedford Way, London, WC1H 0AP UK; 3 Present Address: Google Brain, 1600 Amphitheater Parkway, Mountain View, CA 94304 USA

**Keywords:** Cognitive modeling, Similarity kernel, Psychological embedding, Active learning, Python package

## Abstract

Psychological embeddings provide a powerful formalism for characterizing human-perceived similarity among members of a stimulus set. Obtaining high-quality embeddings can be costly due to algorithm design, software deployment, and participant compensation. This work aims to advance state-of-the-art embedding techniques and provide a comprehensive software package that makes obtaining high-quality psychological embeddings both easy and relatively efficient. Contributions are made on four fronts. First, the embedding procedure allows multiple trial configurations (e.g., triplets) to be used for collecting similarity judgments from participants. For example, trials can be configured to collect triplet comparisons or to sort items into groups. Second, a likelihood model is provided for three classes of similarity kernels allowing users to easily infer the parameters of their preferred model using gradient descent. Third, an active selection algorithm is provided that makes data collection more efficient by proposing comparisons that provide the strongest constraints on the embedding. Fourth, the likelihood model allows the specification of group-specific attention weight parameters. A series of experiments are included to highlight each of these contributions and their impact on converging to a high-quality embedding. Collectively, these incremental improvements provide a powerful and complete set of tools for inferring psychological embeddings. The relevant tools are available as the Python package *PsiZ*, which can be cloned from GitHub (https://github.com/roads/psiz).

## Introduction

In many interactive software systems, it is essential to model an individual’s behavior during a perceptual task. Decision support applications anticipate and adjust for novice perception in order to help novice users arrive at expert-like categorization decisions (Fang & Geman, 2005; Ferecatu & Geman, 2009; Roads & Mozer, 2017). Human-in-the-loop computer vision algorithms utilize a model of human similarity to improve machine categorization performance (Wah et al., 2014). Human category-training applications use cognitive models to predict learning outcomes (e.g., Nosofsky 1986; Kruschke 1992; Love, Medin, & Gureckis, 2004; Nosofsky, Sanders, & McDaniel, 2018). At the core of these applications is the notion of stimulus feature representations and psychological similarity.

The primary objective of this work is to provide a method for *jointly* inferring a multi-dimensional feature representation and a corresponding similarity function. Given a feature representation, a similarity function specifies the degree that responses associated with one stimulus transfer to another (Shepard, 1987; Nosofsky, 1986; Tenenbaum, 1999). The more similar stimuli are, the more likely generalization is to occur. Similarity is based not on external properties of the stimuli, but rather on an individual’s internal representation. We refer to this internal representation—coupled with a similarity function—as a *psychological embedding*.

A mature set of algorithms exists that uses proximity data (e.g., pair-wise similarity ratings) to infer a low-dimensional embedding (Gower, 1966; Torgerson, 1958). These *multidimensional scaling* or*MDS* algorithms can be classified based on two properties: the type of observations used to support inference and the form of the similarity function. In psychology, two dominant types of observations are similarity (dissimilarity) *ratings* (e.g., Torgerson 1952, 1958) and ordinal similarity *rankings* (e.g., Agarwal et al., 2007; Tamuz, Liu, Belongie, Shamir, & Kalai, 2011; van der Maaten & Weinberger 2012). Similarity ratings are elicited by asking participants to rate the similarity between pairs of stimuli on a predefined scale. Similarity rankings are elicited by asking participants to rank stimuli in order of similarity; typically by presenting participants with triplets and asking them to select the pair that is most similar. This work focuses on similarity rankings given their benefits of rater consistency (Demiralp, Bernstein, & Heer, 2014), subject-specific precision (Li, Malave, Song, & Yu, 2016), and cost-effective scalability (Wilber, Kwak, & Belongie, 2014).

The second property of MDS algorithms concerns the form of the similarity function. The similarity function specifies how distance in the embedding space translates to psychological similarity. The least-constrained form assumes only that similarity decays monotonically as a function of distance (Shepard 1962a, 1962b; Kruskal 1968a, 1968b). While being the most flexible, such a similarity function has an increased risk of over-fitting and discounts existing psychological research on stimulus generalization. At the other extreme, similarity is defined as a fixed, parameter- free function (Gower, 1966; Torgerson, 1958). In between these two extremes, similarity is specified as a function with one or more free parameters (Shepard, 1957; Nosofsky, 1985; Tamuz et al., 2011; van der Maaten & Weinberger, 2012). Our work is also situated in between these two extremes, and continues a tradition of using parameterized similarity functions that are well motivated by psychological theory (Shepard 1957, 1958; Nosofsky 1985, 1986).

The primary purpose of this work is to provide a unified set of state-of-the-art tools for individuals interested in inferring psychological embeddings. These tools are collected in a Python package called PsiZ, short-hand for psychological embedding. The Greek letter *Ψ* is commonly associated with psychology, while ***z*** is often used in machine learning to denote a latent feature vector, and ***Z*** ≡{***z***_1_,...,***z***_*n*_} is a matrix containing a collection of vectors. PsiZ unites four facets that can be adjusted to suit the needs of the user. First, observations used for inference can be collected using a variety of different trial configurations (e.g., ranking, clustering). Second, a number of different similarity functions can be used for performing inference, some derived from psychological research and others widely used in machine learning. Additional similarity functions can easily be implemented by the user. Third, the embedding algorithm can be used to infer group-specific attention weights in the same spirit as INDSCAL (Carroll & Chang, 1970). Lastly, high-quality embeddings can be constructed with less data via an active-selection algorithm that intelligently determines which stimulus comparisons should be collected next. The active-selection algorithm is similar to the capabilities provided by the powerful NEXT system (Jamieson, Jain, Fernandez, Glattard, & Nowak, 2015; Rau, Mason, & Nowak, 2016; Sievert et al., 2017). Unlike the more general-purpose NEXT system, PsiZ focuses exclusively on psychological embeddings and aims to provide a comprehensive set of features, classes, and utilities. Each of these facets is described in turn, followed by experiments highlighting the potential benefits to researchers. The relevant tools are available as the Python package *PsiZ*, which can be cloned from https://github.com/roads/psiz. The code used to run the experiments can be cloned from https://github.com/roads/psiz-brm.

## Data collection

**Fig. 1:**
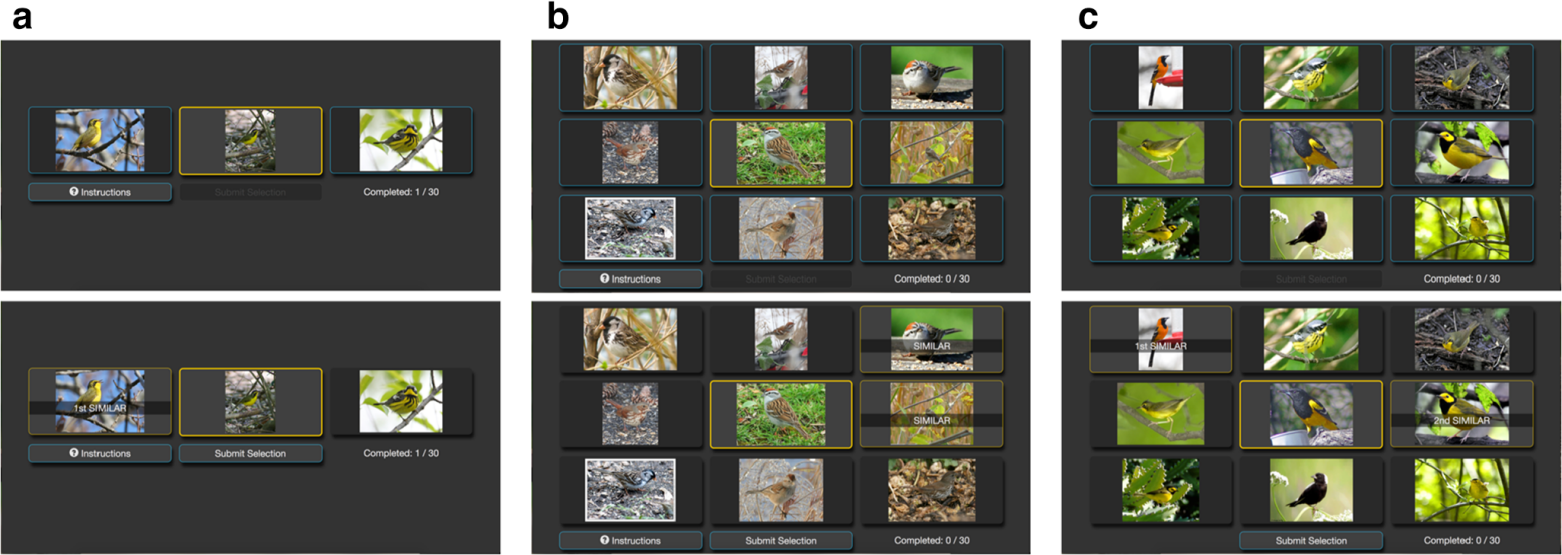
Sample displays shown to subjects. The *center image* is the query stimulus while the surrounding images are the reference stimuli. **a** Given two reference images, subjects select the one reference image that is most similar to the query. **b** Given eight reference images, subjects select the two reference images that are most similar to the query. **c** Given eight reference images, subjects select two reference images in the order of their similarity

Inference of a psychological embedding requires a set of observations, or judged trials. On each trial, subjects compare a *query* stimulus to a set of *reference* images $\mathcal {R}$. In the simplest case, a trial contains two reference images and participants must select the reference image they believe is most similar to the query (Fig. 1a). In addition to this basic trial configuration, PsiZ is designed to work with more complicated configurations—like those that have become popular in the machine learning literature (e.g., Wilber et al.,^2014^; Wah et al., 2014).

Let us consider all the different trial configurations that PsiZ can handle. Since PsiZ allows the configuration to switch on each trial, we denote the configuration parameters of the *i* th trial using the subscript *i*. The set of references $\mathcal {R}_{i}$ can contain 2-8 stimuli. Given a set of references, subjects select a predefined number of reference stimuli that they consider most similar to the query. The set of selected reference images $\mathcal {S}_{i}$, may be 1 to $|{\mathcal {R}_{i}}|-1$. The last configuration parameter determines whether subjects must rank their selection. In the basic case, where $|{\mathcal {R}_{i}}|=2$ and $|{\mathcal {S}_{i}}|=1$, subjects provide triplet similarity judgments (Fig. 1a). In a more complicated scenario, subjects can partition the set of references into a similar and dissimilar group (Fig. 1b). Alternatively, subjects may be asked to rank their selections (Fig. 1c).

In order to simplify the description of the embedding models—as well as the actual code implementation—all observations are assumed to be notated in a specific way. The set of all observations is denoted by $\mathcal {D}$. Assuming that each stimulus has been assigned a unique index, $\mathcal {R}_{i}$ and $\mathcal {S}_{i}$ are sets of stimulus indices on trial *i*. Each judged trial collects one observation. Response information for trial *i*, denoted $\mathcal {D}_{i}$, is coded as a triple, $\mathcal {D}_{i} = \left (q_{i}, \boldsymbol {s}_{i}, \boldsymbol {u}_{i}\right )$, where *q*_*i*_ indicates the index of the query stimulus, ***s***_*i*_ is a row vector comprised of the selected reference stimulus indices, and ***u***_*i*_ is a row vector comprised of the unselected reference stimulus indices. Depending on the trial configuration, the length of $\mathcal {D}_{i}$ will vary. For example, if $|{\mathcal {R}_{i}}|=2$ and $|{\mathcal {S}_{i}}|=1$, $\mathcal {D}_{i}=\left (q_{i}, a_{i}, b_{i} \right )$, where *a*_*i*_ indicates the index of the selected reference stimulus and *b*_*i*_ indicates the index of the unselected reference stimulus. If $|{\mathcal {R}_{i}}|=8$, $|{\mathcal {S}_{i}}|=2$ and subjects make ranked selections, then $\mathcal {D}_{i}=\left (q_{i}, a_{i}, b_{i}, c_{i}, d_{i}, e_{i}, f_{i}, g_{i}, h_{i} \right )$. Now *a*_*i*_ indicates the index of the most similar reference and *b*_*i*_ indicates the index of the second most similar reference, and *c*_*i*_-*h*_*i*_ indicate the remaining unselected references.

PsiZ uses the class psiz.trials.Observations to create a set of judged trials. You can also create a set of unjudged trials using the class psiz.trials.Docket. The initialization format of these classes is shown below. 
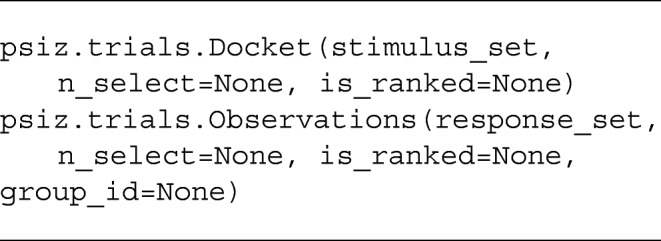


The stimulus set argument for a Docket is a matrix where each row indicates the set of stimuli used in a trial. The first column indicates the query stimulus, while the remaining columns indicate the references. The stimulus set argument for a Docket is also a matrix where each row indicates the responses of a single trial. The columns of the matrix are organized according to formatting described above, i.e., the first column indicates the query, the second column indicates the first selected reference, and so on. By default, both Docket and Observations will assume that n_select= 1 and is_ranked=True. The Observations class also allows you to pass in a group indicator (group_id), which is used when inferring group-specific parameters (described in more detail below).

One way to think about the information contained in the different trial configurations is to consider how many noisy triplet constraints are *implied* by a single trial (Wah et al., 2014). Holding all other factors constant, providing more triplet constraints improves the quality of the inferred solution. In the basic case ($|{\mathcal {R}}|=2$, $|{\mathcal {S}}|=1$), each trial provides one triplet constraint of the form *q* : *a* > *b*, where *a* is the reference stimulus that was selected as more similar to the query *q*. More generally, for unordered selections, each display yields $|{\mathcal {S}}|\left (|{\mathcal {R}}| - |{\mathcal {S}}|\right )$ triplet constraints. For ordered selections, each trial yields $|{\mathcal {S}}|\left (|{\mathcal {R}}| - |{\mathcal {S}}|\right )+ \binom {|{\mathcal {S}}|}{2}$ triplet constraints. The best trial configuration will depend in part upon the time needed to complete a single trial and the reliability of subject responses (Wilber et al., 2014).

## Embedding model

Given a set of observations, the goal is to infer a psychological embedding, which consists of two parts: a multi-dimensional feature representation and a corresponding similarity function. To improve conceptual clarity, the free parameters representing and the feature representation (***Z***) and the parameters controlling the similarity function (***𝜃***) are written separately. Observed behavior is linked to the parameters of interest (***Z***, ***𝜃***) using a generative model. The generative model describes how the observed behavior is generated from the parameters of interest. Given a set of observations $\mathcal {D}$, the likelihood of the observations given the model parameters is:

1 $$ \mathcal{L} = \prod\limits_{i} p\left( \mathcal{D}_{i} | \boldsymbol{Z}, \boldsymbol{\theta} \right) $$

It should be noted that the likelihood allows researchers to combine different trial configurations since each trial is assumed to contribute independently to the likelihood. In the remainder of this section, we walk through the generative model, starting with the distance function.

### Distance function

To start, we assume the feature representation ***Z*** is composed of points in a *D*-dimensional space. Following decades of psychological research (e.g., Nosofsky^1985^), we further assume that distance between points is computed using the weighted Minkowski distance:

2 $$ \|{\boldsymbol{z}_{m} - \boldsymbol{z}_{m}}\|_{\rho,\boldsymbol{w}_{k}} = \left( \sum\limits_{j=1}^{D}w_{j}|{z_{m,j} - z_{n,j}}|^{\rho}\right)^{\frac{1}{\rho}}, $$

where *w*_*j*_ ≥ 0 and ${\sum }_{j=1}^{D} w_{j} = D$. Note that the weights sum to *D*, so that when all the weights are equal, i.e., *w*_*j*_ = 1, we recover the standard (unweighted) Minkowski distance. The arguments ***z***_*m*_ and ***z***_*n*_ indicate two arbitrary feature representations. The parameter *ρ* controls the type of distance (e.g., *ρ* = 2 results in Euclidean distance).

The weights correspond to attention weights and allow the model to capture differences in how individuals or groups attend to different dimensions in the psychological embedding. When inferring a population-level model (i.e., there is only one group), all the weights are set to one. In the single group case, setting the weights to one does not eliminate any degrees of freedom. Since the weights are jointly inferred with the embedding vectors, the vectors can adjust themselves during inference to incorporate any stretching or shrinking of the dimensions. In the most general case, these weights are allowed to vary by individual or group. In the remainder of this work, we use ***w***_*k*_ to indicate the attention weights of group *k*.

### Similarity function

Equipped with a distance function, we assume that psychological similarity is described by a parameterized function based on the weighted Minkowski distance:

3 $$ s\left( \boldsymbol{z}_{m}, \boldsymbol{z}_{n} \right) = f\left( \|{\boldsymbol{z}_{m} - \boldsymbol{z}_{n}}\|_{\rho,\boldsymbol{w}_{k}}\right). $$

Given two embedding points ***z***_*k*_ and ***z***_*l*_, the similarity function $s\left (\boldsymbol {z}_{k}, \boldsymbol {z}_{l} \right )$ returns a value, where 0 indicates that the two points are maximally dissimilar. Three popular parameterizations assume that similarity decays exponentially (Shepard 1957, 1958; Nosofsky 1985, 1986), inversely with respect to distance (Agarwal et al., 2007; Tamuz et al., 2011), or according to the Student’s-*t* kernel (van der Maaten & Weinberger, 2012).

In addition to these approaches, there are two other popular approaches: classical MDS (Gower, 1966; Torgerson, 1958) and standard non-metric MDS (Shepard 1962a, 1962b; Kruskal 1968a, 1968b). Classical MDS assumes an identity relationship between distances and *dissimilarity*. While there are methods for converting similarity to dissimilarity (e.g., by subtracting dissimilarity from a constant), introducing these transformations quickly moves away from a faithful implementation of the approach. Standard non-metric MDS only assumes a monotonic relationship between distance and similarity. Typically, this monotonic relationship is determined using isotonic regression. Isotonic regression results in a piece-wise linear function with multiple discontinuities, which creates problems for gradient-based inference.

#### Exponential-family kernel

Integrating various psychological models (e.g., Jones, Love, & Maddox, 2006; Jones, Maddox, & Love, 2006; Nosofsky 1986; Shepard 1987) into their most general form, we obtain:

4 $$ s\left( \boldsymbol{z}_{m}, \boldsymbol{z}_{n} \right) = \exp\left( -\beta \|{\boldsymbol{z}_{m} - \boldsymbol{z}_{n}}\|_{\rho,\boldsymbol{w}_{k}}^{\tau}\right) + \gamma, $$

where *β*, *τ*, and *γ* are free parameters that control the gradient of generalization. Since the most common parameter settings result in a Laplacian kernel (*τ* = 1, *ρ* = 2, *γ* = 0) and Gaussian kernel (*τ* = 2, *ρ* = 2, *γ* = 0), we refer to this class of similarity functions as the *exponential-family* kernel.

#### Student’s-*t* kernel

Although substantial psychological evidence supports the idea that individuals use an exponential similarity function, other similarity functions have been used with success. In machine learning, a popular similarity function is the Student’s-*t* kernel (van der Maaten & Weinberger, 2012):

5 $$ s\left( \boldsymbol{z}_{m}, \boldsymbol{z}_{n} \right) = \left( 1 + \frac{\|{\boldsymbol{z}_{m} - \boldsymbol{z}_{n}}\|_{2,\boldsymbol{w}_{k}}^{2}}{\alpha} \right)^{-\frac{\alpha + 1}{2}}. $$

A primary advantage of the Student’s-*t* kernel is that it has a heavy tail. The heavy tail is advantageous during inference because it provides a signal to the inference algorithm to continue pushing similar points together and dissimilar points apart.

#### Inverse distance kernel

A second similarity function that has been widely used in machine learning is the inverse distance kernel (Agarwal et al., 2007; Tamuz et al., 2011):

6 $$ s\left( \boldsymbol{z}_{m}, \boldsymbol{z}_{n} \right) = \frac{1}{\mu + \|{\boldsymbol{z}_{m} - \boldsymbol{z}_{n}}\|_{\rho,\boldsymbol{w}_{k}}^{\tau}} $$,

where *μ* and *τ* are free parameters that govern similarity. The free parameter *τ* serves the same role as in the exponential-family kernel. The parameter *μ* is included to make the approach numerically stable.

#### Heavy-tailed kernel

By itself, the Student’s-*t* kernel lacks the flexibility of the exponential kernel. By generalizing the Student’s-*t* kernel with additional free parameters, one obtains a heavy-tailed kernel with comparable flexibility to the exponential kernel:

7 $$ s\left( \boldsymbol{z}_{m}, \boldsymbol{z}_{n} \right) = \left( \kappa + \|{\boldsymbol{z}_{m} - \boldsymbol{z}_{n}}\|_{\rho,\boldsymbol{w}_{k}}^{\tau}\right)^{-\alpha}. $$

### Selection function

The last component is the selection function, which specifies how perceived similarity is converted into observed behavior. Given a similarity function, the likelihood of subject selections are modeled in the same spirit as Luce’s ratio of strengths formulation (Luce, 1959) and classic similarity choice models (Shepard 1957, 1958; Nosofsky^1985^, ^1986^). The basic principle is that the probability of selecting a given reference is proportional to the similarity between the query and that reference. For example, when subjects make only one selection ($|{\mathcal {R}_{i}}| \in \left [2,8\right ]$, $|{\mathcal {S}_{i}}|=1$), the likelihood of the observed outcome is,

8 $$ p\left( \mathcal{D}_{i} | \boldsymbol{Z}, \boldsymbol{\theta} \right) = \frac{s\left( \boldsymbol{z}_{q}, \boldsymbol{z}_{a} \right)}{{\sum}_{r \in \mathcal{R}_{i}} s\left( \boldsymbol{z}_{q}, \boldsymbol{z}_{r} \right)}. $$

This basic principle is expanded following the rules of probability in order to describe more complicated trial configurations. For example, when a trial requires participants to select two (unranked) references ($|{\mathcal {R}_{i}}| \in \left [3,8\right ]$ and $|{\mathcal {S}_{i}}|=2$),

9 $$ \begin{array}{@{}rcl@{}} p\left( \mathcal{D}_{i} | \boldsymbol{Z}, \boldsymbol{\theta} \right) &=& \frac{s\left( \boldsymbol{z}_{q}, \boldsymbol{z}_{a} \right)}{{\sum}_{r \in \mathcal{R}_{i}} s\left( \boldsymbol{z}_{q}, \boldsymbol{z}_{r} \right)}\frac{s\left( \boldsymbol{z}_{q}, \boldsymbol{z}_{b} \right)}{{\sum}_{r \in \mathcal{R}_{i\neg a}} s\left( \boldsymbol{z}_{q}, \boldsymbol{z}_{r} \right)} \\&&+ \frac{s\left( \boldsymbol{z}_{q}, \boldsymbol{z}_{b} \right)}{{\sum}_{r \in \mathcal{R}_{i}} s\left( \boldsymbol{z}_{q}, \boldsymbol{z}_{r} \right)}\frac{s\left( \boldsymbol{z}_{q}, \boldsymbol{z}_{a} \right)}{{\sum}_{r \in \mathcal{R}_{i\neg b}} s\left( \boldsymbol{z}_{q}, \boldsymbol{z}_{r} \right)}. \end{array} $$

The selection function is similar when a participant is required to select and rank two reference,

10 $$ p\left( \mathcal{D}_{i} | \boldsymbol{Z}, \boldsymbol{\theta} \right) = \frac{s\left( \boldsymbol{z}_{q}, \boldsymbol{z}_{a} \right)}{{\sum}_{r \in \mathcal{R}_{i}} s\left( \boldsymbol{z}_{q}, \boldsymbol{z}_{r} \right)}\frac{s\left( \boldsymbol{z}_{q}, \boldsymbol{z}_{b} \right)}{{\sum}_{r \in \mathcal{R}_{i \neg a}} s\left( \boldsymbol{z}_{q}, \boldsymbol{z}_{r} \right)}. $$

## Inference procedure

Equipped with a likelihood a set of observations, it is now possible to perform inference. The PsiZ package leverages the TensorFlow Python library (Abadi et al., 2015) to perform gradient-based inference on the log-likelihood of the data given the model parameters:

11 $$ \max\limits_{\boldsymbol{Z}, \boldsymbol{\theta}} \sum\limits_{i} \log p\left( \mathcal{D}_{i} | \boldsymbol{Z}, \boldsymbol{\theta} \right). $$

The burden of solving this optimization problem is almost completely removed, allowing researchers to call a few high-level methods in order to achieve their goals. For example, it is simple to infer a psychological embedding using an exponential similarity function. 
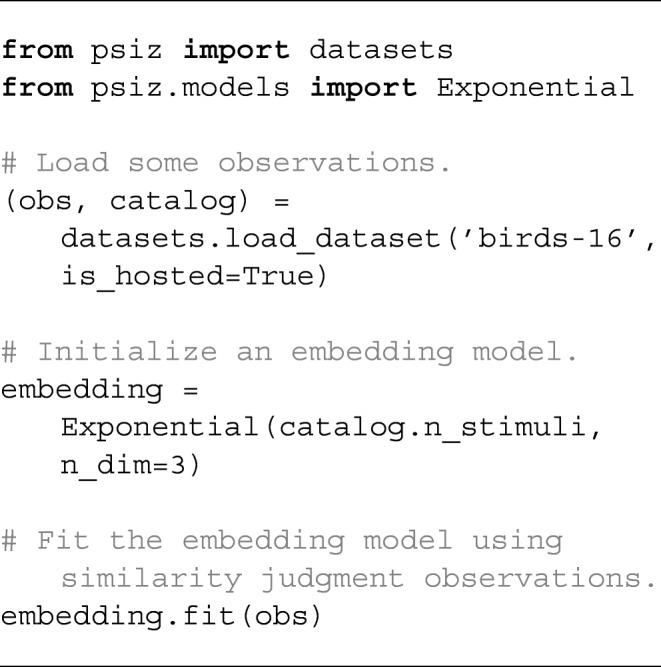


The above example loads a set of observations, initializes a three-dimensional embedding model, and then fits the model to the loaded observations. While this example requests a three-dimensional embedding, the researcher must decide which dimensionality is appropriate for their problem. The PsiZ package includes a separate procedure that researchers can use to help them decide on the appropriate dimensionality. 
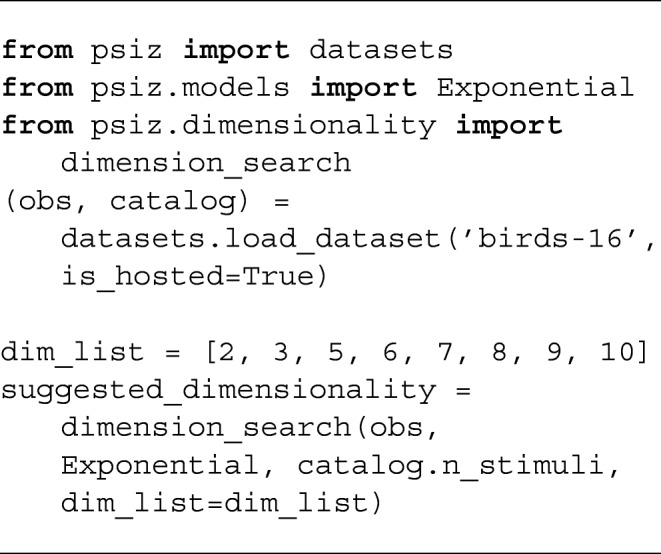


The dimension_search procedure first partitions the provided observations into a train and hold-out set. For each of the candidate dimensionalities provided by the user, an embedding is fit using the training set and is evaluated using the validation set. The procedure continues to try embeddings with a larger dimensionality until validation loss stops improving. When validation loss is worse for the current dimensionality under consideration, the procedure terminates and returns the dimensionality that resulted in the last observed improvement.

When inferring an embedding, it is important to assess whether a sufficient number of observations have been collected. If an insufficient number of observations have been collected, the inferred embedding is much more likely to model noise rather than actual behavior. One method for determining if enough data have been collected is to perform a simple convergence analysis. To perform a convergence analysis, the observations are split up into multiple partitions. A separate embedding is inferred using observations from an increasing number of partitions. Each embedding is compared to the previous embedding by computing a Pearson correlation coefficient between the two embeddings. If a sufficient amount of data have been collected, then adding more data should not change the inferred embedding and the correlation score should be high. PsiZ provides a simple function called assess_convergence for performing a convergence analysis. 
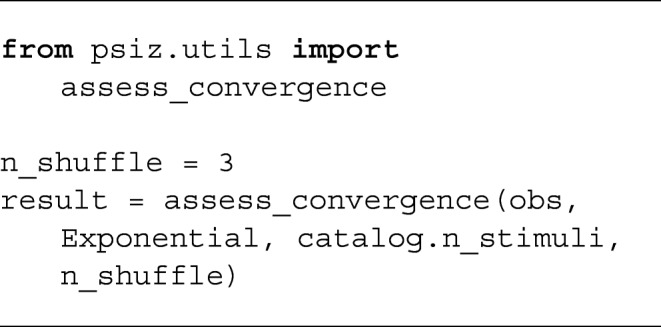


In the above example, n_shuffle indicates the number of times the analysis should be repeated. In between each analysis, the data is shuffled, giving a different set of partitions each time.

## Intelligent trial selection

The final aspect of this work focuses on intelligently selecting trials to collect maximally informative data. A simple strategy is to randomly select images for each trial, with the sole constraint that all images be unique. Although a reasonable approach, random displays have a drawback. Imagine for the moment that judged displays have been collected using all but one stimulus. Ideally, the next trial shown to a participant would include that unused stimulus. More generally, the embedding procedure will be less confident about the position of some images in an embedding. An active-selection approach constructs trials that have the best chance of minimizing uncertainty associated with the embedding. Active selection proceeds via multiple iterations of generating trials and collecting the corresponding observations. This component is heavily inspired by previous active selection research (Tamuz et al., 2011), but has been generalized to handle arbitrary trial configurations and uses a different set of heuristics.

### Formalizing uncertainty

The uncertainty of an embedding point’s location is formally characterized using a posterior distribution,

12 $$ p\left( \boldsymbol{z}_{k} | \mathcal{D}, \boldsymbol{Z}_{\neg k}, \boldsymbol{\theta}\right) \propto p\left( \mathcal{D} | \boldsymbol{Z}, \boldsymbol{\theta}\right) p\left( \boldsymbol{z}_{k} | \boldsymbol{Z}_{\neg k}, \boldsymbol{\theta}\right). $$

For simplicity, we assume that the prior distribution of the embedding points is characterized using a Gaussian distribution

13 $$ p\left( \boldsymbol{z}_{k} | \boldsymbol{Z}_{\neg k} \boldsymbol{\theta}\right) \sim \mathcal{N}\left( {\mu}, \boldsymbol{\Sigma} \right). $$

The likelihood is the same as previously described and predicts how participants select references given a particular query,

14 $$ p\left( \mathcal{D} | \boldsymbol{Z}, \boldsymbol{\theta}\right). $$

The posterior distribution is approximated by sampling from the joint distribution using Gibbs sampling. Since the posterior distribution has a Gaussian prior, elliptical slice sampling (Murray, Adams, & MacKay, 2010) can be used to sample points in a relatively efficient manner. In effect, the sampling procedure produces a set of points for each stimulus. If the distribution of points is tightly clustered, then there is relatively low uncertainty about the position of that stimulus. If the distribution is wide, then there is relatively high uncertainty about the location of the stimulus in the embedding. For clarity, the posterior samples are denoted using a three-dimensional tensor *ζ* such that $ {\zeta }^{(s)}_{k}$ indicates the *s*’th sample of the *k*’th stimulus, a *d*-dimensional vector corresponding to a point in the embedding space. The matrix *ζ*^(*s*)^ can be thought of as a sampling snapshot of the entire embedding.

### Maximizing information gain

To maximize information gain, we need to compute the expected information gain for a candidate trial. Given a candidate trial ***c***, the expected information gain is equal to the mutual information,

15 $$ I\left( \boldsymbol{Z}; Y | {\mathcal{D}}, \boldsymbol{c} \right) = H\left( \boldsymbol{Z} | {\mathcal{D}} \right) - H\left( \boldsymbol{Z} | {\mathcal{D}}, Y, \boldsymbol{c} \right), $$

where *Y* is a discrete random variable indicating all possible outcomes when the candidate trial is shown to a participant. For example, if the candidate trial displays two references and participants must select one reference, then there are two possible outcomes. The first term indicates the entropy (i.e., uncertainty) associated with the current embedding. The second term indicates the expected entropy of the embedding if we collect an observation for the candidate trial. Since we would like to minimize entropy associated with the embedding, we are looking for a candidate trial such that $H\left (\boldsymbol {Z} |  {\mathcal {D}} \right ) > H\left (\boldsymbol {Z} |  {\mathcal {D}}, Y, \boldsymbol {c} \right )$ and information gain is positive.

Since ***Z*** is a continuous variable, computing information gain appears non-trivial. Fortunately, the computation can be simplified by exploiting the identity of mutual information (i.e., *H*(*A*) − *H*(*A*|*B*) = *H*(*B*) − *H*(*B*|*A*)) and using our previously obtained samples taken from the posterior distribution in order to approximate the integrals. After all modifications and approximations, the equation for information gain becomes,

16 $$ \begin{array}{@{}rcl@{}} I\left( \boldsymbol{Z};Y | \mathcal{D}, \boldsymbol{c} \right) & = & \!- \sum\limits_{i=1}^{M} P\left( y_{i} | \mathcal{D},\! \boldsymbol{c} \right) \log P\left( y_{i} | \mathcal{D}, \boldsymbol{c} \right) \\&&\!+ \frac{1}{N} {\sum\limits_{s}^{N}} {\sum\limits_{i}^{M}} p (y_{i} | {\zeta}^{(s)},\! \mathcal{D} ) \log p(y_{i} | {\zeta}^{(s)}, \mathcal{D} ) ,\\ \end{array} $$

where *M* indicates the number of possible outcomes associated with the candidate trial and *N* is the number of samples being used to approximate the integral.

### Heuristic search procedure

For simple scenarios, it is possible to evaluate all candidate trials in order find the trial that maximizes expected information gain. Unfortunately, for most scenarios, particularly those involving larger stimulus sets, exhaustive search becomes prohibitively expensive. As an alternative, we employ a two-stage heuristic search strategy. In the first stage, a query stimulus is stochastically selected based on its relative uncertainty. In the second stage, a candidate set of references is stochastically selected based on their similarity to the query stimulus. This process is repeated until the desired number of trials have been created. Ideally, the embedding would be updated to take into account observations for the new trial. In practice, multiple trials can be generated at once by limiting the number of times a particular stimulus can be used as a query.

In the first stage, relative uncertainty is determined by summing the Kullback–Leibler divergence between the stimulus of interest and all other stimuli. Intuitively, this prioritizes stimuli that exhibit high uncertainty in the embedding. However, not all uncertainty is equivalent from the perspective of constraining the embedding. Rather, high uncertainty stimuli with close neighbors should be prioritized over high uncertainty stimuli with distant neighbors. The asymmetric nature of Kullback–Leibler divergence is also exploited in this heuristic. Given two stimuli, one that has high uncertainty and one that has low uncertainty, only the stimulus with high uncertainty should be given higher priority. Once the relative uncertainty has been determined for all stimuli, a query is stochastically selected proportional to its relative uncertainty such that higher uncertainty stimuli are more likely to be chosen as query stimuli.

In the second stage, a set of candidate references are selected based on similarity using the current best estimate of the similarity function. The candidate references are selected stochastically such that more similar neighbors are more likely to be chosen. In effect, this heuristic biases the reference set to include stimuli that are close neighbors of the query stimulus. If all the reference stimuli are excessively dissimilar from the query stimulus, the corresponding similarity judgments will not provide much information. When few observations have been collected, this heuristic prioritizes unevaluated stimuli.

## Experiment and model recovery simulations

### Experiment 1: kernel comparison

The following experiment compares the ability of three different similarity kernels to predict human similarity judgments. The exponential-family kernel is motivated by psychological theory, while the inverse distance kernel, Student’s-*t* kernel, and heavy-tailed kernel are largely motivated by common practice in machine learning.

#### Methods

##### Participants

A population of 342 participants were recruited from Amazon Mechanical Turk and paid at a rate of approximately $6.00 per hour.

##### Materials

**Fig. 2:**
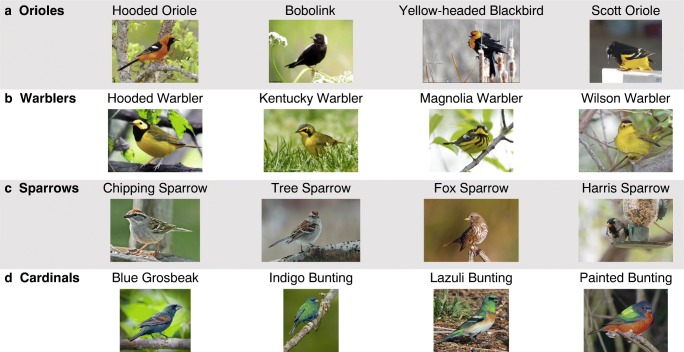
Example stimuli of the different bird species used in this work. Each row contains four similar bird species, each of which belongs to the same or similar taxonomic family. The images shown in this figure were drawn from the set of 208 images used in the experiments

A small dataset of 16 species of birds was assembled from the CUB 200 image dataset (Wah, Branson, Welinder, Perona, & Belongie, 2011). Species were chosen such that there were four groups of birds composed of four similar-looking species, roughly corresponding to four taxonomic families (Fig. 2a–d). For each species, we selected 13 images, yielding a total of 208 unique images. Images were selected and cropped such that each image displayed a single bird, the bird was clearly visible, the image was of a good resolution, and no text was present.

##### Procedure

Similarity judgments were collected during short, 10-min sessions via a web-based application deployed on Amazon Mechanical Turk. Each 10-min session used one of two possible trial configurations. Participants either saw trials with two references and selected the most similar reference (2-choose-1) or saw eight references and selected two references in a ranked order (8-choose-2). During a 2-choose-1 session, participants saw between 60 and 120 trials. The number of displays varied in order to calibrate each session to be approximately 10 min. During an 8-choose-2 session, participants saw 30 trials. Participants were allowed to complete more than one 10-min session. Collectively, participants judged 7520 2-choose-1 trials and 8772 8-choose-2 trials. All judged trials were combined to create a single dataset of observations ($\mathcal {D}$).

The collected similarity judgments were used in a tenfold, subject-stratified cross-validation procedure in order to evaluate the capabilities of an exponential-family kernel, an inverse distance kernel, a heavy-tailed kernel, and a Student’s-*t* kernel. Similarity judgments were partitioned into ten roughly equal folds such that each fold had the same proportion of 2-choose-1 and 8-choose-2 trials. For each fold, the dimensionality was estimated using the dimension_search routine. Once a dimensionality was selected, an embedding was inferred using nine folds as training data. The remaining fold was used as a validation set. For each fold, the validation loss (i.e., negative log-likelihood) and validation accuracy was recorded. Accuracy was computed differently for each trial configuration. For 2-choose-1 configurations, accuracy was determined by computing the proportion of time the model correctly predicted the chosen reference (i.e., top-1 accuracy). For 8-choose-2 configurations, there are 56 possible outcomes. The prediction of an 8-choose-2 was graded as correct if the actual outcome was among the top five most probable outcomes (top-5 accuracy). Since individuals may perceive similarity differently and individuals themselves may not be consistent, we do not expect to infer embeddings with zero loss or perfect accuracy.

#### Results

**Fig. 3:**
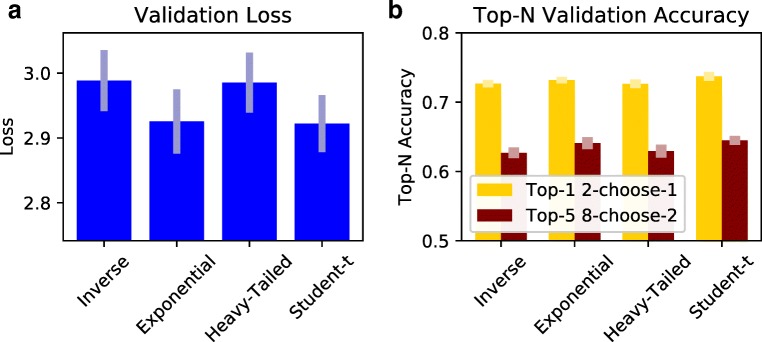
Model fitting results using a tenfold cross-validation procedure for four different similarity kernels: inverse, exponential, heavy-tailed, and Student’s-*t*. Validation results are shown for **a** validation loss (i.e., negative log-likelihood) and **b** top-n validation accuracy. Top-1 accuracy is used evaluate 2-choose-1 trials while top-5 accuracy is used to evaluate 8-choose-2 trials. All *error bars* indicate standard error of the mean across the ten folds

The primary goal of the model comparison is to determine which model is best able to predict unseen human similarity judgments. The results are presented in Fig. 3. During the cross-validation procedure, the dimension_search procedure selected a dimensionality that varied between 2 and 4. The modal dimensionality for the inverse, exponential, heavy-tailed, and Student’s-*t* model was 4, 3, 2, and 3, respectively. Significance tests use a Bonferroni corrected alpha value of .05 (.008 corrected). Focusing on validation loss, pairwise *t* tests of the tenfold cross-validation validation procedure reveal that the differences between the inverse kernel (*M* = 2.99, *SD* = 0.14), exponential-family kernel (*M* = 2.93, *SD* = 0.15), the heavy-tailed kernel (*M* = 2.99, *SD* = 0.14), and the Student’s-*t* kernel (*M* = 2.92, *SD* = 0.13) are all non-significant. Likewise, pairwise *t* tests of top-*N* accuracy were all non-significant.

To ensure that the null results were not merely a result of insufficient data, a convergence analysis was performed using the assess_convergence function. When splitting the data into ten partitions and performing three different restarts, the Pearson correlation between the similarity matrices of an embedding that uses all of the data and an embedding that uses nine partitions of the data is very high (*ρ* = .95). The high correlation value suggests that the inferred embeddings are consistent and a sufficient amount of data have been collected.

#### Discussion

The three kernels appear equally capable of predicting human similarity judgments. For domains that are similar to the set of birds used here, it is likely reasonable to use either kernel. One advantage of using the exponential-family kernel is that many computational models of human category learning also use an exponential-family kernel. By assuming an exponential-family kernel, the resulting psychological embedding could be integrated with a category learning model (e.g., Shepard 1957, 1958; Nosofsky 1985, 1986; Roads, Xu, Robinson, & Tanaka, 2018).

Given a domain as complicated as birds, it may be surprising that the dimensionality search choose relatively low dimensionality embeddings. While birds unquestionably involve a plethora of features, the embedding algorithm is constrained by three factors. First, the chosen bird dataset does not span the entire space of bird features. If a particular type of feature variability is not represented in the dataset, it is unlikely to be captured in the inferred embedding. Second, the inferred embeddings represent nonlinear manifolds of a higher-dimensional feature space. Consequently, a single embedding dimension is unlikely to model a single visual feature, but will capture a mixture of features. Lastly, the embeddings are constrained by the number of observations that are available. Each additional dimension adds an additional degree of freedom for *every* embedding vector. Without sufficient data, these additional degrees of freedom will lead to model overfitting and poor generalization. Under ideal conditions, an infinite number of observations would be collected in order to allow the dimensionality search procedure to consider higher dimensional spaces.

### Simulation 1: data collection strategies

Having compared different kernels, we turn to the issue of comparing different strategies for collecting observations. The goal is to determine the strategy that results in the highest-quality embedding at the lowest cost. Since the primary cost of collecting similarity judgments is paying participants for their time, we evaluate different collection strategies based on how many worker hours are required to reach a given quality level.

Data collection strategies are evaluated along two dimensions. First, the trial itself can take on many different configurations. Second, trials can be generated randomly or via active selection. The different collection strategies are evaluated using simulations of human similarity judgments.

#### Methods

##### Participants

No new participants were recruited for this experiment. All similarity judgments collected for the previous experiment were re-used to infer a ground-truth model of human behavior.

##### Materials

The experiment used the same set of 208 bird images as the previous experiment.

##### Procedure

Three different collection strategies were evaluated using simulated human responses. The first collection strategy presented trials containing two references, where simulated participants selected one reference. The content of the trials was chosen randomly, subject to the constrain that a single image could not appear more than once on a trial (random 2-choose-1). The second collection strategy presented trials containing eight references and required participants to select two references, in ranked order. The particular images for each trial were chosen randomly (random 8-choose-2). The last strategy used an 8-choose-2 trial configuration, but selected the trial content using active selection (active 8-choose-2).

Simulated responses were generated by treating a fitted psychological embedding as a generative model of human behavior (i.e., a virtual subject). Once a psychological embedding predicts the probability of all possible response outcomes for a particular trial (see “Selection function”), a specific response is generated by stochastically sampling from the possible outcomes. To ensure that the simulated responses mirror human behavior, an exponential-family psychological embedding was fitted to all human similarity judgments described in the previous experiment (7520 2-choose-1 trials and 4733 8-choose-2 trials). The fitted model served as a virtual subject and the ground-truth psychological embedding by which other models were evaluated.

Each collection strategy is used to generate trials, collect observations, and infer a strategy-specific psychological embedding. The quality of a strategy-specific embedding is determined by comparing its predictions to those of the ground-truth embedding. The critical predictions of a psychological embedding can be summarized by generating a corresponding pair-wise similarity matrix *S*. The element *s*_*ij*_ indicates the similarity between the *i* th and *j* th stimulus. The predictions of a strategy-specific and ground-truth psychological embedding can be compared by computing the Pearson correlation coefficient between the respective similarity matrices. When computing the Pearson correlation, we only use the upper diagonal portion of the matrix less the diagonal elements, since the matrix is symmetric and the diagonal elements indicate self-similarity. If the strategy-specific embedding has successfully modeled the ground-truth embedding, the Pearson correlation will be high.

Each strategy-specific embedding was inferred using a different number of trials in order to map out how the number of trials affects the quality of the inferred embedding. Starting with an initial set of observations, additional observations were added in an incremental fashion. Each strategy-specific embedding was evaluated based on how many worker hours it took to reach a Pearson correlation of .95. Since there are two sources of stochasticity (trial generation and response simulation), five separate runs were conducted for each strategy. For each run, random 2-choose-1, random 8-choose-2, and active 8-choose-2 were seeded with 500, 50, and 50 trials, respectively. For all strategies, the seed trials had their content generated randomly. During active selection, 40 trials (each with a unique query image) were generated per round. In between every round, the posterior distribution of the embedding points was updated, while holding constant the parameters of the similarity function. Every fifth round, the parameters of the similarity function were updated. For simplicity, all inference is performed assuming a dimensionality of three—matching the dimensionality of the ground-truth embedding.

#### Results

From the actual human data, it is clear that a 2-choose-1 display (*M* = 4.73, *SD* = 11.18) and 8-choose-2 (*M* = 13.07, *SD* = 23.49) display require different amounts of time to complete (*t*(12251) = 26.41, *p* < 0.001). The number of trials is converted to worker hours based on the median human response time of the 2-choose-1 (median 3.06 s) and 8-choose-2 (median 8.98 s) trials. Since the human response times include dramatic outliers, median response times provide an appropriate measure of central tendency.

**Fig. 4:**
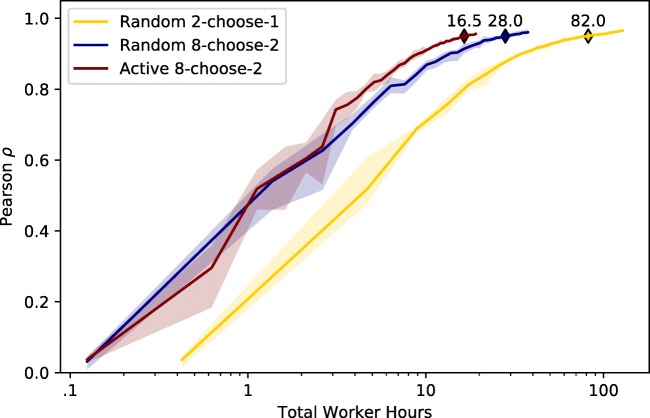
Results of Simulation 1. Each *line* indicates a different collection strategy. Since cost is determined by total number of worker hours needed, the quality of the inferred embeddings is plotted with respect to worker hours. Each *line* indicates the mean between five independent simulation runs. The *shaded regions* indicate the maximum and minimum envelope across runs

The simulation results for three different collection strategies are presented in Fig. 4. The simulation results show that random 8-choose-2 (M = 28.0 h) is more efficient than random 2-choose-1 (M = 82.0 h) in reaching a Pearson correlation of .95. For the same embedding quality, only about 34% of the worker hours are necessary when using randomly selected 8-choose-2 versus 2-choose-1 trials. The results also reveal that active 8-choose-2 (M = 16.0 h) is more efficient than either random strategy. When using an 8-choose-2 trial configuration, active selection requires about 57% of the worker hours compared to random selection.

#### Discussion

Assuming reliability of responses is in accordance with our simulations, using 8-choose-2 displays is more cost-effective than 2-choose-1 displays, allowing a high-quality embedding to be inferred at nearly a third of the cost. If the goal is to obtain a psychological embedding using the most effective trial configuration, the 8-choose-2 trial configuration is a good way to save money. These results replicate the findings of Wilber et al., (2014), except with a proper likelihood model.

Additional savings are achieved when switching to a strategy that uses active selection. The benefit of active selection appears to be greatest when the quality of the inferred embedding is starting to asymptote. Without much data, active selection behaves similarly to random selection. In effect, the active selection procedure is highly uncertain about *all* embedding points and the chosen displays provide the same amount of information at randomly generated displays. As data accumulate, active selection is able to focus on uncertain stimuli, allowing the inferred embedding to reach asymptote more quickly. According to these simulations, active selection provides an efficient and cost-effective way to obtain high-quality embeddings.

### Simulation 2: group-specific attention weights

In the final simulation, we demonstrate how the embedding procedure is capable of inferring group-specific attention weights in a similar spirit to MDS procedure INDSCAL (Carroll and Chang, 1970). Attention weights assume that all agents use the same feature space, but may differ in how important they view each dimension. For example, an expert birdwatcher may place more weight on the color of feathers surrounding a bird’s eye, while a novice may place very little weight on this feature. Since the development of INDSCAL, many cognitive models have captured individual and group differences using attention weights (e.g., Nosofsky 1984, 1986; Kruschke 1992; Love et al.,^2004^).

In addition to demonstrating the ability to learn group-specific attention weights, this simulation also demonstrates how inferring a shared embedding has the potential to reduce the cost of collecting data. The demonstration uses a shared set of fictitious stimuli and two simulated groups. These two groups can be likened to novices and experts. Inspired by novice and expert attention differences with musical notes (Shepard, 1982), we assume a scenario where novices pay attention to one set of feature dimensions, while experts attend to a complementary set of feature dimensions.

#### Methods

##### Participants

No human participants were used in this experiment. All observations were simulated.

##### Materials

A fictitious set of 100 stimuli was used in this experiment. The stimuli coordinates were drawn from a four-dimensional Gaussian distribution with zero mean and a spherical covariance matrix of 0.03 (equal variance along each dimension).

##### Procedure

Following the design of Simulation 1, we assume a known ground-truth psychological embedding. In contrast to the previous experiment, this embedding is not based on actual human behavior, but assumes that a set of stimuli are distributed in a four-dimensional space. Furthermore, it is assumed that novices focus on the first two dimensions (**w** = [1.8,1.8,.2,.2]) while experts focus on the last two dimensions (**w** = [.2,.2,1.8,1.8]) when judging similarity. Novice and expert responses are simulated using the respective attention weights.

Multiple-group inference is examined using two conditions: a naive approach (independent) and an information-sharing approach (shared). The naive approach is to infer an independent embedding for each group. There are two primary disadvantages with this approach. First, independently inferred embeddings are not directly comparable, since MDS solutions exhibit rotation and scale invariance. Second, independently inferred embeddings will not be able to leverage any mutual information between the observations of the two groups. The shared condition infers a shared psychological embedding with group-specific attention weights.

The quality of an inferred embedding is evaluated in the same manner as Simulation 1, with a small twist. Since there are two groups, there are group-specific similarity matrices: a novice similarity matrix and an expert similarity matrix. The quality of an inferred embedding is determined by comparing each group-specific similarity matrix to the corresponding ground-truth similarity matrix.

The independent and shared condition require different considerations when deciding how to collect observations. In the independent condition, each group is treated independently and the researcher collects whatever number of observations are necessary for each group. In the shared condition, the researcher could collect an equal number of novice and expert observations. Since it is assumed that there is mutual information between the two groups, the researcher can choose to collect an imbalanced set of observations that optimizes some external utility function. For example, it is often the case that compensating experts costs more than compensating novices. If the external utility function is to minimize cost, the researcher can collect a larger proportion of novice observations. The best proportion will be determined by the particular circumstances of the researcher. As a proof of concept, we consider the case where novice observations are collected approximately 74% of the time.

Using five independent runs, we determine how many expert worker hours are necessary to reach psychological embeddings that correctly capture novice and expert behavior with at least a .95 Pearson correlation. Worker hours are estimated using the same conversion values used in Simulation 1.

#### Results

**Fig. 5:**
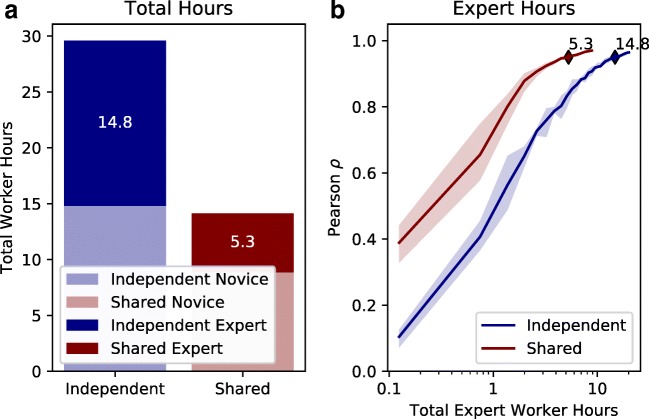
Results of Simulation 2. **a** Total worker hours for the independent and shared condition. The results indicate an average of five independent runs. **b** A breakdown of expert-specific convergence as a function of expert worker hours. Each *line* indicates a different simulated scenario evaluating how many expert worker hours are necessary. The *blue line* indicates the number of expert worker hours necessary to reach criterion when a independent psychological embedding is inferred for each group. The *red line* indicates the number of expert worker hours necessary to reach criterion when a shared psychological embedding is inferred. Each line indicates the mean between five independent simulation runs. The *shaded regions* indicate the maximum and minimum envelope across runs

The simulation expert-specific results for the two different conditions are presented in Fig. 5. When inferring an expert-specific psychological embedding for the independent condition, approximately 14.8 expert worker hours are required to reach criterion. Inferring a novice-specific psychological embedding requires 14.8 novice worker hours to reach criterion (not shown in the figure). When inferring a shared embedding, criterion can be met for both groups using 8.9 novice worker hours and 5.3 expert worker hours. From the perspective of total worker hours, the independent condition requires 29.6 worker hours while the shared condition requires 14.2 worker hours.

#### Discussion

Fewer total worker hours are required when inferring a single psychological embedding with group-specific attention weights than when inferring two independent embeddings. It is notable that the shared condition only requires about half the total worker hours as the independent condition. The large difference is likely driven by three factors. First, a shared embedding involves fewer free parameters in total. Second, a shared embedding can take advantage of any mutual information between the novice and expert groups. Lastly, inference likely struggles to determine the location of stimuli along the feature dimensions that receive little attention weight. By combining observations from subjects that have complementary attention weights, it becomes easier to determine the location of each stimulus in the psychological embedding. Combined, these factors make the shared condition a clear winner.

In addition to fewer total worker hours, a shared psychological embedding can reach criterion for both experts and novices by using relative fewer *expert* hours. Since experts are typically paid more for their time (and expertise), reducing the required number of expert worker hours can substantially reduce the financial burden of collecting data. It is possible that more extreme savings can be achieved by shifting more of the inference burden onto novice observations. The above analysis assumed that novice and experts complete trials in the same amount of time. However, novices and experts may differ on their throughput. One possibility is that experts would be faster given their ability to make quick fine-grained judgments about their domain of expertise (Tanaka & Taylor, 1991).

## Conclusions

Psychological embeddings are useful in many domains of research. Despite the substantial progress that has been made, a unified and coherent set of tools has been slow to emerge. This work presents the key aspects of a publicly available Python package that makes it easy for researchers to infer their own psychological embeddings. In an effort to make the tools as useful as possible, the algorithms have been designed to handle a variety of trial configurations and handle inference of group-specific attention weights. In addition, the package includes an active selection routine to help researchers get the most out of their budget. While these facets were discussed in the context of visual similarity, the software package can work with similarity judgment based on other modalities.

To accompany the description of the algorithm, three experiments demonstrated the various ways the package can be used. Experiment 1 demonstrated how different similarity kernels can easily be compared, allowing the researcher to select the one that makes the most sense for their project. Simulation 1 highlighted how different collection strategies can make data acquisition more cost-effective. In particular, active selection combined with 8-choose-2 trial configurations beat out the other options. Lastly, Simulation 3 illustrated how group-specific attention weights can be inferred using a single model—potentially reducing the cost of collecting data. In isolation, the results presented in this work make incremental contributions on four different fronts. As a whole, a meaningful contribution is made by providing a complete top-to-bottom software package.
